# Genetic, Molecular, and Pathogenic Characterization of the H9N2 Avian Influenza Viruses Currently Circulating in South China

**DOI:** 10.3390/v11111040

**Published:** 2019-11-08

**Authors:** Hailiang Sun, Jiate Lin, Zhiting Liu, Yanan Yu, Meihua Wu, Shuo Li, Yang Liu, Yaling Feng, Yuqian Wu, Mingliang Li, Peirong Jiao, Kaijian Luo, Ming Liao

**Affiliations:** College of Veterinary Medicine, South China Agricultural University, Guangzhou 510642, China; hsun@scau.edu.cn (H.S.); wcnjzrn@163.com (M.L.); prjiao@scau.edu.cn (P.J.); kjluo@scau.edu.cn (K.L.)

**Keywords:** influenza A virus, H9N2, replication, pathogenicity, receptor, pigs

## Abstract

The prevalence and variation of the H9N2 avian influenza virus (AIV) pose a threat to public health. A total of eight viruses isolated from farmed poultry in South China during 2017–2018 were selected as representative strains for further systematic study. Phylogenetic analyses indicated that these prevalent viruses belong to the Y280-like lineage and that the internal genes are highly similar to those of recently circulating human H7N9 viruses. The receptor-binding assay showed that most of the H9N2 isolates preferentially bound to the human-like receptor, increasing the risk of them crossing the species barrier and causing human infection. Our in vitro, multi-step growth curve results indicate these viruses can effectively replicate in mammalian cells. Infection in mice showed that three viruses effectively replicated in the lung of mice. Infection in swine revealed that the viruses readily replicated in the upper respiratory tract of pig and effectively induced viral shedding. Our findings suggested that the H9N2 AIVs circulating in poultry recently acquired an enhanced ability to transmit from avian to mammalians, including humans. Based on our findings, we propose that it is essential to strengthen the efforts to surveil and test the pathogenicity of H9N2 AIVs.

## 1. Introduction

H9N2 subtype avian influenza viruses (AIVs) have been mainly circulating in Asia [1,2]. Based on the hemagglutinin (HA) gene, H9N2 viruses can be classified into two distinct lineages, the North American lineage and the Eurasian lineage. The Eurasian lineage could be further divided into three major sub-lineages: G1-like, Y280-like/BJ/94-like, and Y439-like [3], of which the G1-like viruses were enzootic in Southeast and South Asia and the Middle East, Y280-like/BJ/94-like viruses were prevalent in China, and Y439-like viruses were circulating in Korea [4,5,6]. In China, the first H9N2 virus was isolated in Guangdong in 1994, and the G1-like, Y280-like, and BJ/94-like viruses were the predominant lineages in poultry throughout the mid-1990s [1,7]. From 2000 onwards, the BJ/94-like viruses were gradually replaced by F/98-like viruses [8]. H9N2 viruses continue to circulate in poultry, even though an inactivated vaccine has been applied in poultry flocks since 1998 [9,10]. The H9N2 viruses generated several genotypes in poultry through extensive reassortments between different lineages from 1994 to 2016 [11,12]. The continuing dynamic evolution and reassortment of H9N2 viruses will make these genotypes increasingly diverse.

H9N2 AIVs cause great economic loss by reducing egg production and through lethal coinfection with other pathogens in poultry, even though H9N2 AIVs exhibit low avian pathogenicity [5,9,13]. Furthermore, H9N2 AIVs have been confirmed as donors of internal genes to highly pathogenic AIVs with pandemic potential. The six internal genes of H5N1 HPAIV that caused the Hong Kong influenza outbreak in 1997 were all from H9N2 AIVs [14]. The H7N9, H10N8, and H5N6 viruses were first detected in China in 2013 and since then have been responsible for much human mortality (their six internal genes were also donated by H9N2 AIVs) [15,16,17,18,19,20,21]. That H9N2 AIVs are continuing to circulate and evolve will further increase the risk of them acting as gene donators to new AIVs. 

Besides circulating in poultry, H9N2 AIV can directly transmit to humans. Infectious cases of H9N2 AIVs in humans were reported as early as around the year 2000 [22,23]. In recent years, increasing numbers of human infection cases have been reported [24,25,26,27]. The serological survey revealed that the antibody rate was 1.3–1.4% in the regular population and reached as much as 15% among poultry workers [22,28,29,30,31]. Additionally, H9N2 AIVs have been reported to infect domestic pigs in Hong Kong and mainland China [32,33,34,35]. Virus neutralization results indicate that pigs in southeastern China have been infected with H9 influenza viruses from as early as 1998 [36]. Hemagglutinin inhibition (HI) results have revealed that pigs throughout China were infected with H9N2 viruses during 2008–2013, with positive serum rates as high as 4.6%–4.87% [37,38]. There is increasing evidence indicating that H9N2 AIV infections in humans and pigs are not rare.

H9N2 acquired multiple amino acid adaptation mutations to transmit from avian to mammalians [12]. The H9N2 viruses recently circulating in poultry pose an increasing threat to public health. The aims of this study were (1) to analyze the molecular characteristics and phylogenetic relationship of the viruses, (2) to detect the replication ability of the viruses in different mammalian cells, (3) to characterize the preference binding affinity of the viruses, and (4) to detect the pathogenicity of the viruses to pigs.

## 2. Materials and Methods

### 2.1. Cells and Pigs

The Madin-Darby canine kidney (MDCK), human lung adenocarcinoma epithelial (A549), swine testicle (ST), and porcine kidney (PK-15) cells were stored in our lab. The porcine alveolar macrophage cells (3D4/21) were purchased from LMAI Bio (Shanghai, China). The human bronchial epithelioid cells (HBE) were purchased from OTWO biotech incorporation (Shenzhen, China). The 6-week-old female SPF mice were purchased from Tianqin biotech incorporation (Changsha, China). The 3-week-old healthy pigs were purchased from a pig farm in Heyuan city in Guangdong province and were housed in pens. The pigs were confirmed serologically negative for influenza, brucellosis, and pseudorabies by HI assays and enzyme-linked immunosorbent assay (ELISA).

### 2.2. Swabs Collection and Virus Isolation

Six-hundred-thirty oropharyngeal and cloacal swabs were collected from live bird markets and chicken slaughterhouses between 2017 and 2018 in Guangdong province, of which 280 swabs were collected from chickens, 220 swabs were collected from ducks, and 130 swabs were collected from geese. The total RNA of swabs was extracted using an RNAfast200 purification kit (Fastagen Biotech, Shanghai, China) according to the manufacturer’s instructions. Reverse transcription polymerase chain reaction (RT-PCR) was performed using the Uni12 primer (AGCAAAAGCAGG). The matrix protein (M) gene was amplified using specific primers [39], and the PCR products were then detected by electrophoresis. M gene-positive swab samples were inoculated into 9-day-old chicken embryos. Viruses were detected using a hemagglutination assay at 48 h post-inoculation, and subtypes were detected using the HI assay. H9 subtype viruses were stored in a −80 °C freezer.

### 2.3. Sequencing and Phylogenetic Analysis

RNA of the H9 subtype viruses was extracted and used for RT-PCR. Then, the eight segments of the viruses were amplified using specific primers [39]. PCR products were purified using the Gel Extraction Kit D2500 (Omega Bio-Tek, Guangzhou, China) and sent to Shanghai Invitrogen Biotechnology Co. for sequencing. Sequencing data were compiled with the SeqMan program of Lasergene7. A phylogenetic tree of the H9N2 influenza A virus was generated by the Maximum Likelihood method using the MEGA 7 software (Sinauer Associates, Inc., Sunderland, MA, USA).

### 2.4. Viral Replication Kinetics in Cells

The in vitro replication characteristics of the eight H9N2 viruses were detected by infection of MDCK, A549, HBE, PK-15, ST, and 3D4/21 cells. A H1N2 subtype swine influenza virus, SW19/16, was used as control. The plaque-forming unit (PFU) of these viruses was detected in MDCK cells according to the previous study [40]. When the confluence of cells cultured in 12-well plates was 90%, the number of cells in each well was counted by a cell counter. Then, the MDCK cells were infected with H9N2 viruses in a multiplicity of infection (MOI) of 0.001, and the A549, PK-15, ST, and 3D4/21 cells were infected with these viruses in an MOI of 0.01. The supernatant of the cell was discarded at 2 h post-infection (hpi) and was washed three times using PBS, and then cultured with 1 mL of Opti-MEM I Reduced Serum Medium (Thermo Fisher Scientific, Asheville, NC, USA). The supernatants of cells were harvested at 12, 24, 36, 48, 60, and 72 hpi, respectively. The titration of supernatant was performed in MDCK cells.

### 2.5. Receptor-Binding Assay

The receptor-binding properties of H9N2 AIVs were detected using a solid-phase binding assay, modified from a previous study [41]. Briefly, polystyrene Universal-Bind microplates (Corning, New York, USA) were coated with 100 µL streptavidin (PuriMag Biotech, Xiamen, China) and diluted with phosphate citrate buffer at a concentration of 10 µg/mL at 37 °C for 12 to 24 h until dry. Next, the plates were washed three times with PBST (phosphate-buffered saline containing 0.05% Tween-20) and incubated with α-2, 3-siaylglycopolymer or α-2, and 6-siaylglycopolymer (GlycoTech, Inc., Gaithersburg, MD, USA), which were serially two-fold diluted with PBS (0.78 to 100 ng/100 µL) at 4 °C for 24 h. Next, the plates were washed three times with PBS and incubated with a monoclonal antibody against H9 subtype AIV diluted with PBS at a concentration of 0.4 nmol/mL at 4 °C for 5 h. After being washed three times with PBST, the plates were incubated with horseradish peroxidase (HRP)-conjugated goat anti-mouse antibody (Bioworld Technology, Nanjing, China) at a concentration of 100 ng/mL at 4 °C for 2 h. Next, the plates were washed three times with PBST and incubated with TMB (3,3’,5,5’-Tetramethylbenzidine) (Solarbio, Beijing, China) at room temperature for 10 min. Then, 0.05 mL of H_2_SO_4_ (0.5 mole/L) was added to the plates. The optical density at 450 nm was determined in a plate reader.

### 2.6. Pathogenicity in Mice

Eighty 6-week-old female BALB/c mice were randomly divided into ten groups and housed under specific pathogen-free conditions. In group one, eight mice were anesthetized with isoflurane and intranasally inoculated with 100 μL PBS as control. Excepting the SW19/16 virus was used at a dose of 10^5^ EID_50_ due to its low viral titers, in the other groups, mice were anesthetized and intranasally inoculated with corresponding influenza viruses at a dose of 10^6^ EID_50_ in 100 μL, respectively. At 3 dpi, three mice in each group were euthanized and brains, spleens, kidneys, and lungs were collected. The viral titers were titrated in MDCK cells. The remaining five mice in each group were continuing to be monitored and weighed until 14 dpi. Mice were euthanized and sera were collected at 14 dpi.

### 2.7. Infection of Pigs

Thirteen healthy 3-week-old pigs that were serologically negative for influenza, brucellosis, and pseudorabies were randomly divided into three groups. In the treatment group, five pigs were intranasally inoculated with CK93/17 at a dose of 10^7^ EID_50_ in 1 mL of PBS at zero days post-inoculation (dpi), and in the physical contact group, five pigs were housed together with treatment pigs at 1 dpi. As a control group, three pigs were intranasally inoculated with 1 mL PBS. All pigs were monitored, and the temperatures were detected for 14 days. Nasal wash was collected from all pigs from 1 to 10 dpi by a followed method. Pigs were anesthetized with Xylazine Hydrochloride Injection purchased from Hanhe Incorpration (Qingdao, China) at a dose of 0.2 mL/kg. Then the nostrils of pigs were washed with 3 mL PBS by pipet and the nasal wash was collected in the dish. Two infected pigs, one contact pig, and one control pig were euthanized at 3 dpi. One infected pig, two contact pigs, and one control pig were euthanized at 5 dpi. Turbinates, tracheas, and lungs were collected. The serum was collected from pigs at 7 and 14 dpi, respectively. The remaining pigs were euthanized at 14 dpi.

### 2.8. Ethics Animal Handling

Experiments involving mice and pigs were conducted in compliance with the principles of the Basel Declaration and recommendations of the approved guidelines of the Experimental Animal Administration and Ethics Committee of South China Agricultural University (SCAUADL2018-010; 12 October, 2017). The protocol (SCAUADL2018-010) was approved by the Experimental Animal Administration and Ethics Committee of South China Agricultural University.

## 3. Results

### 3.1. H9N2 Viruses Form a Novel Genotype

**Figure 1:**
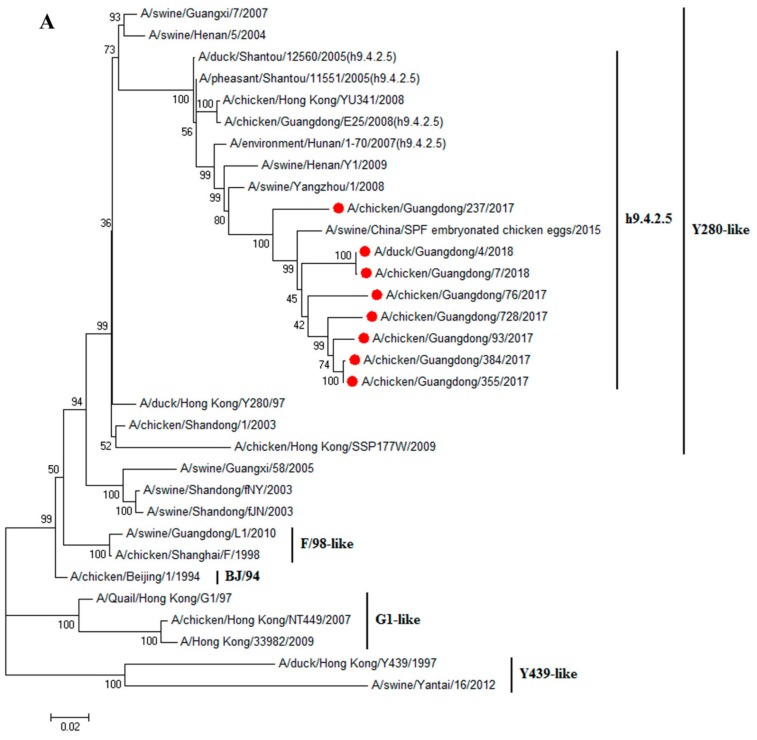

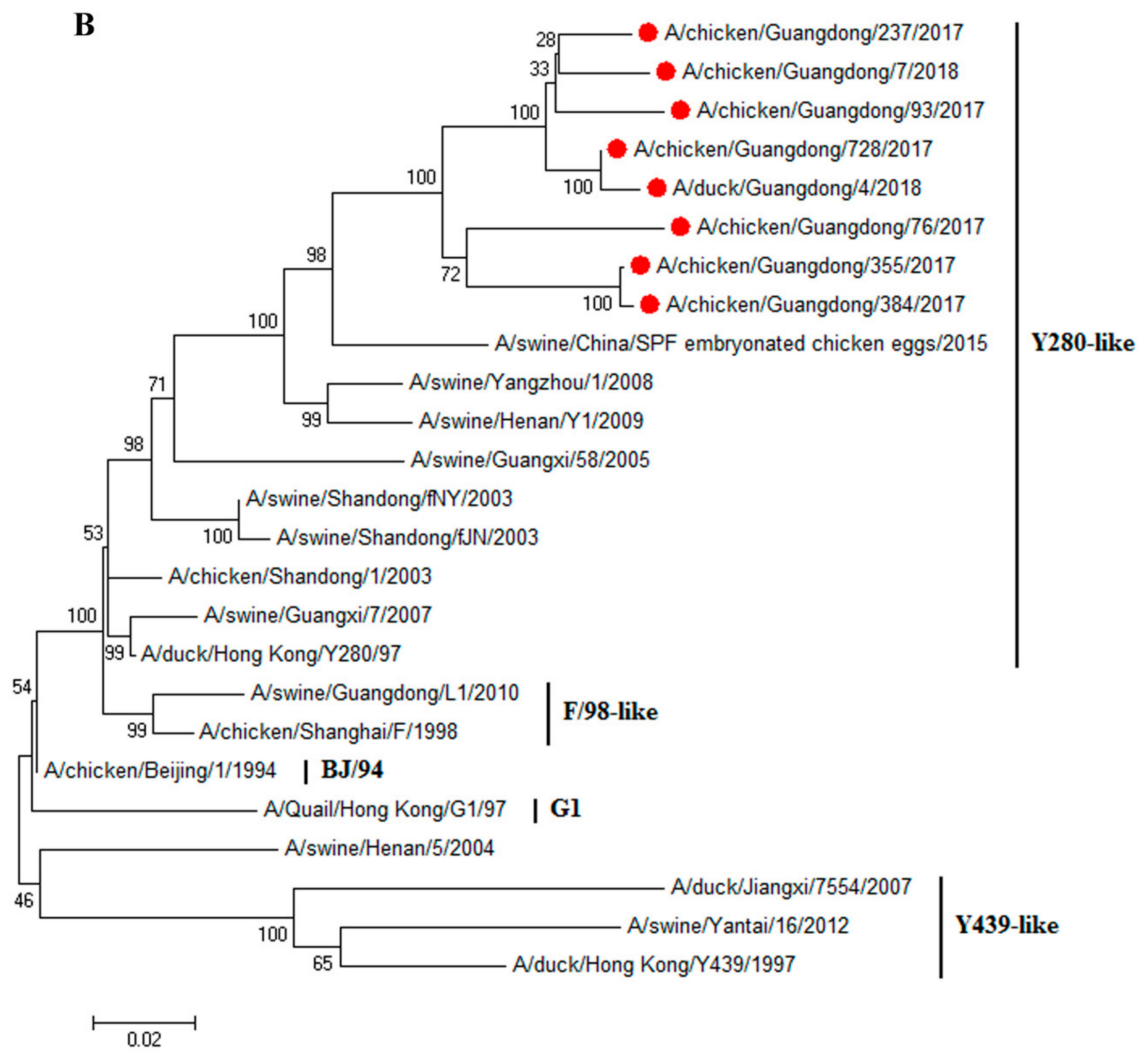

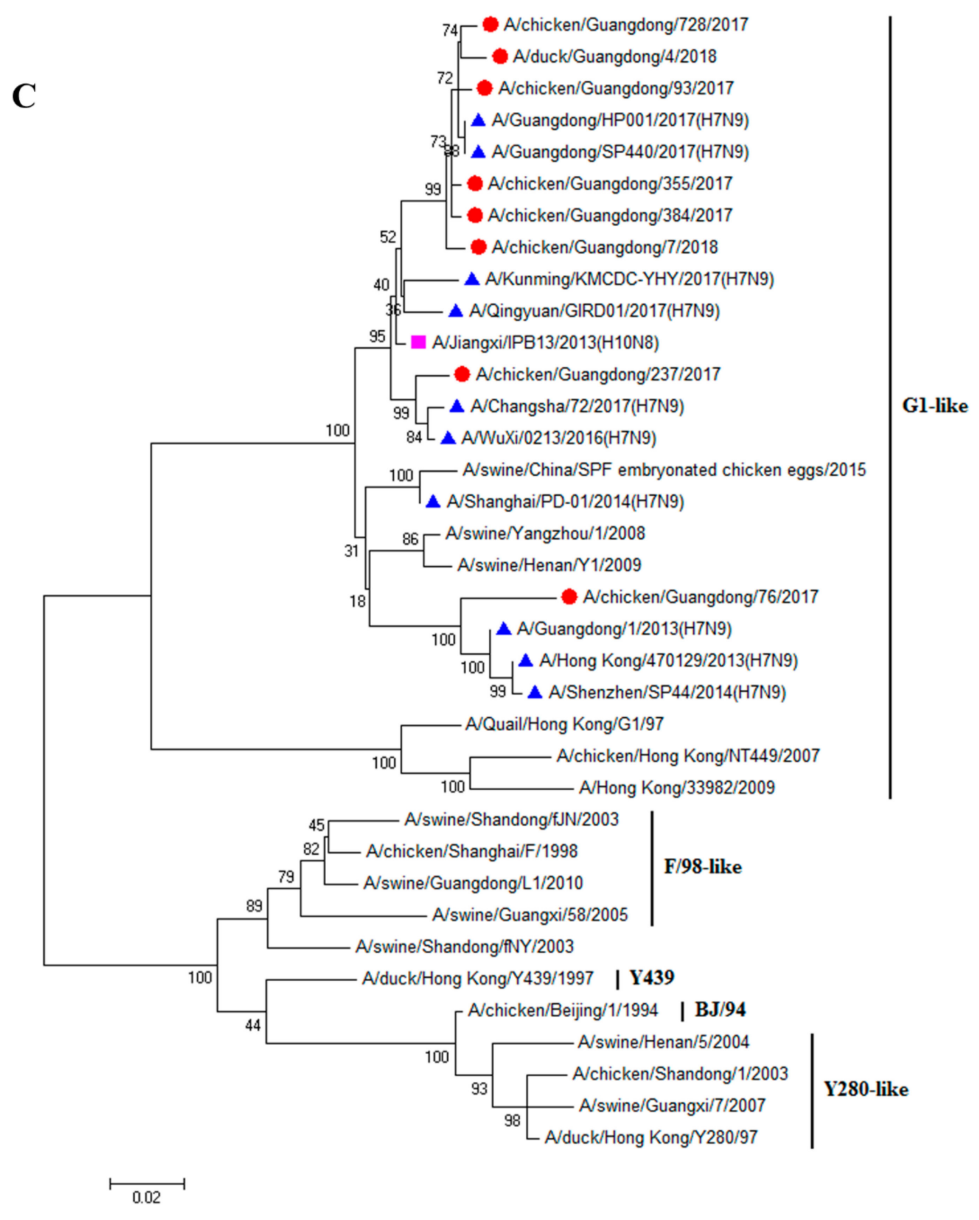

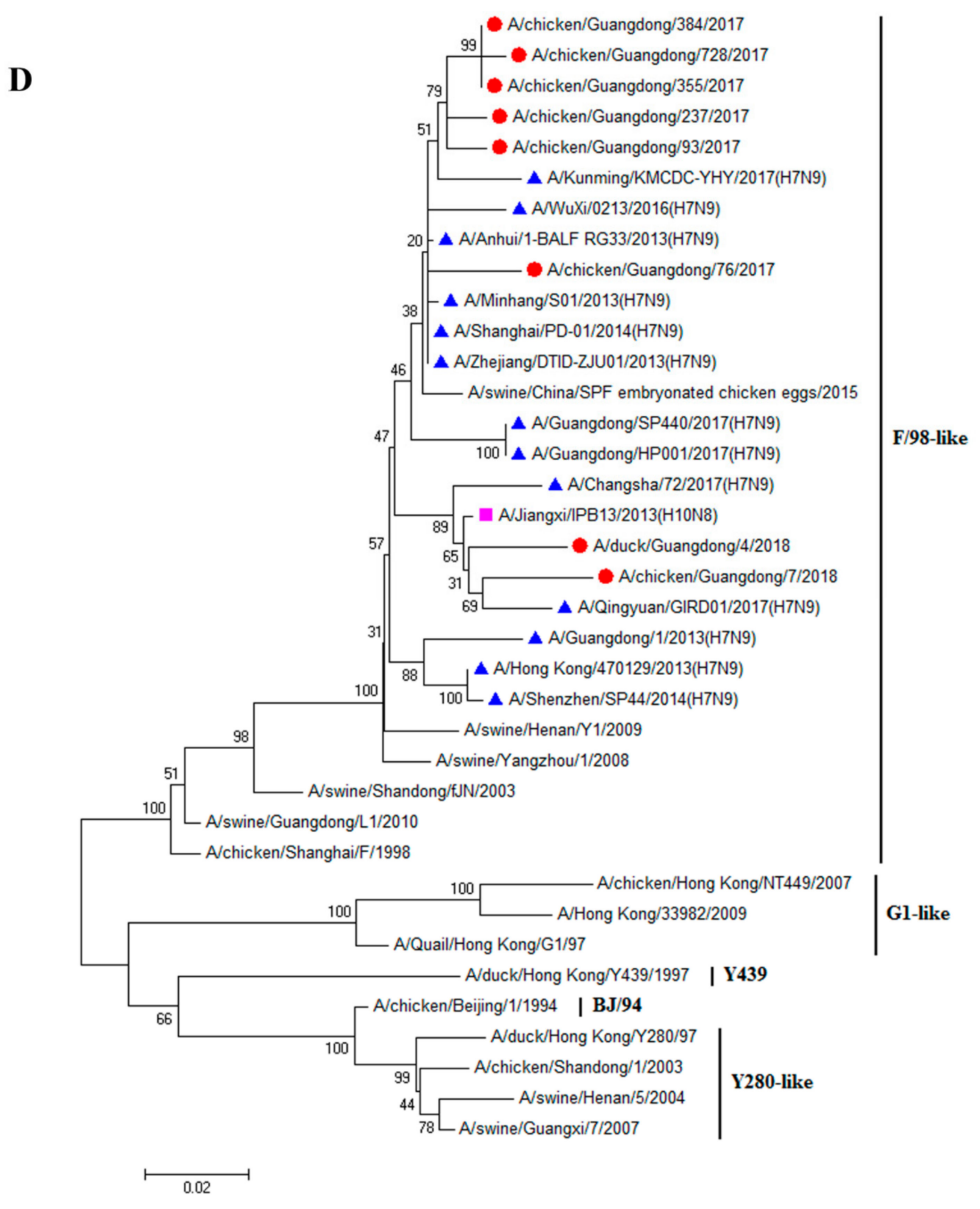

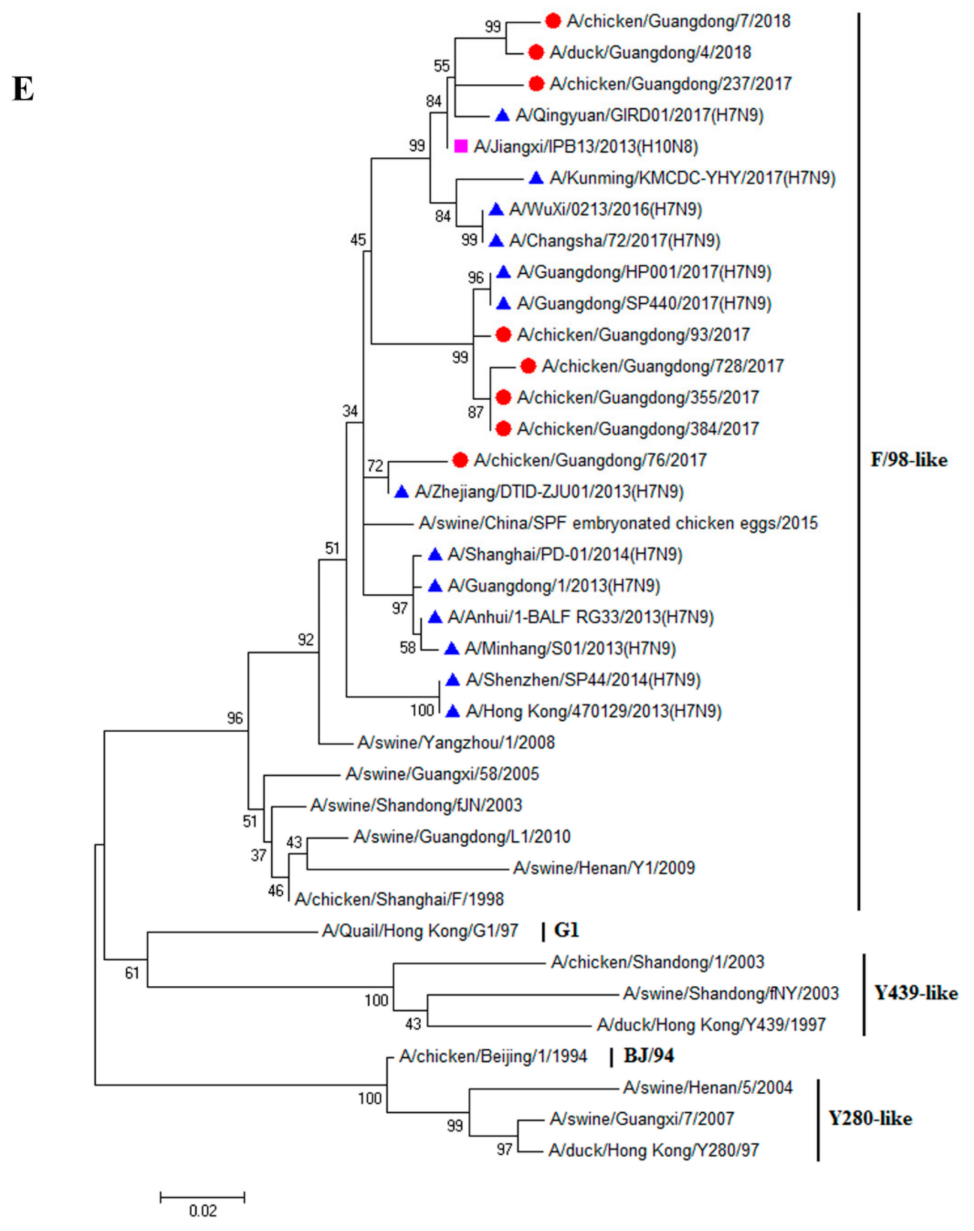

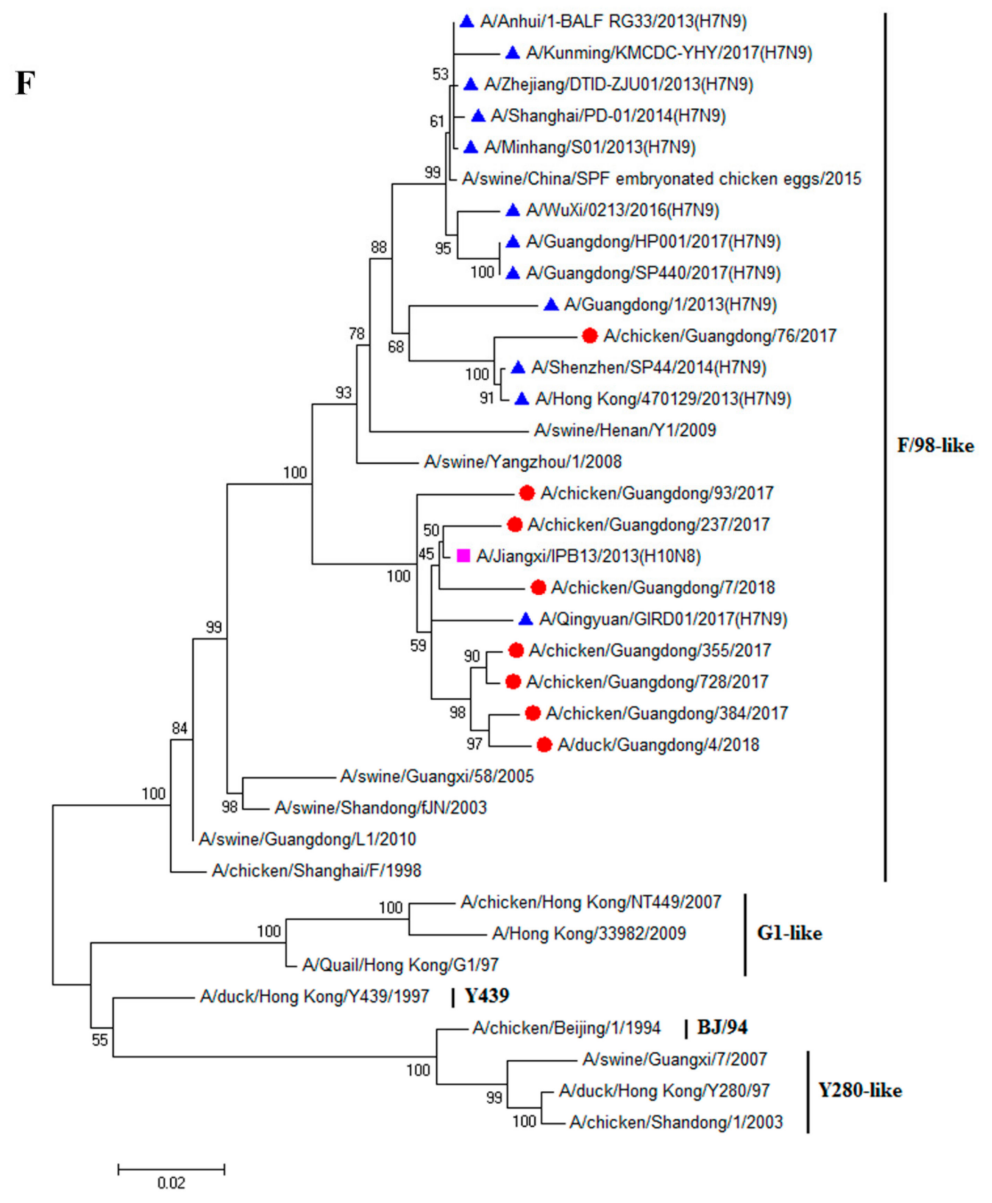

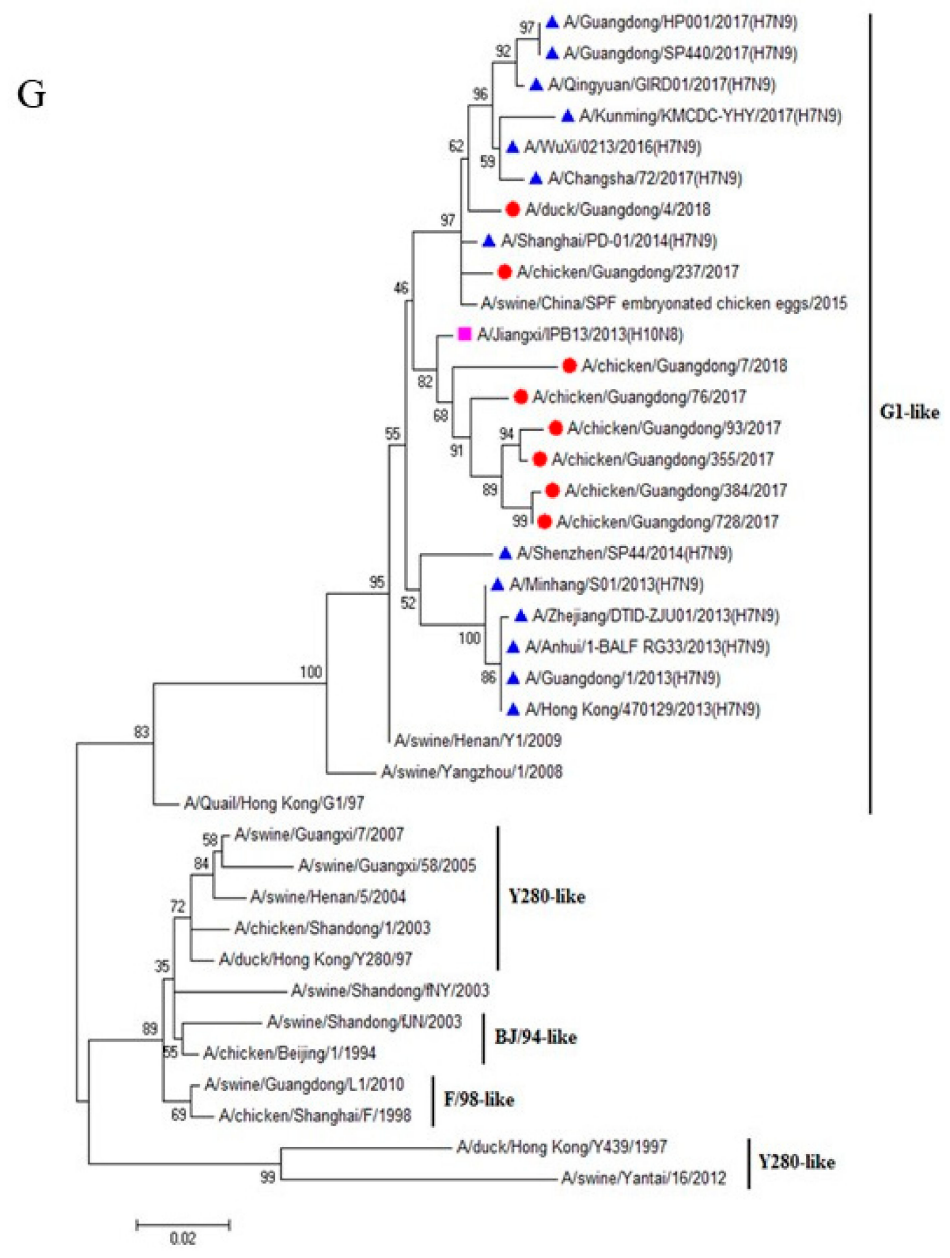

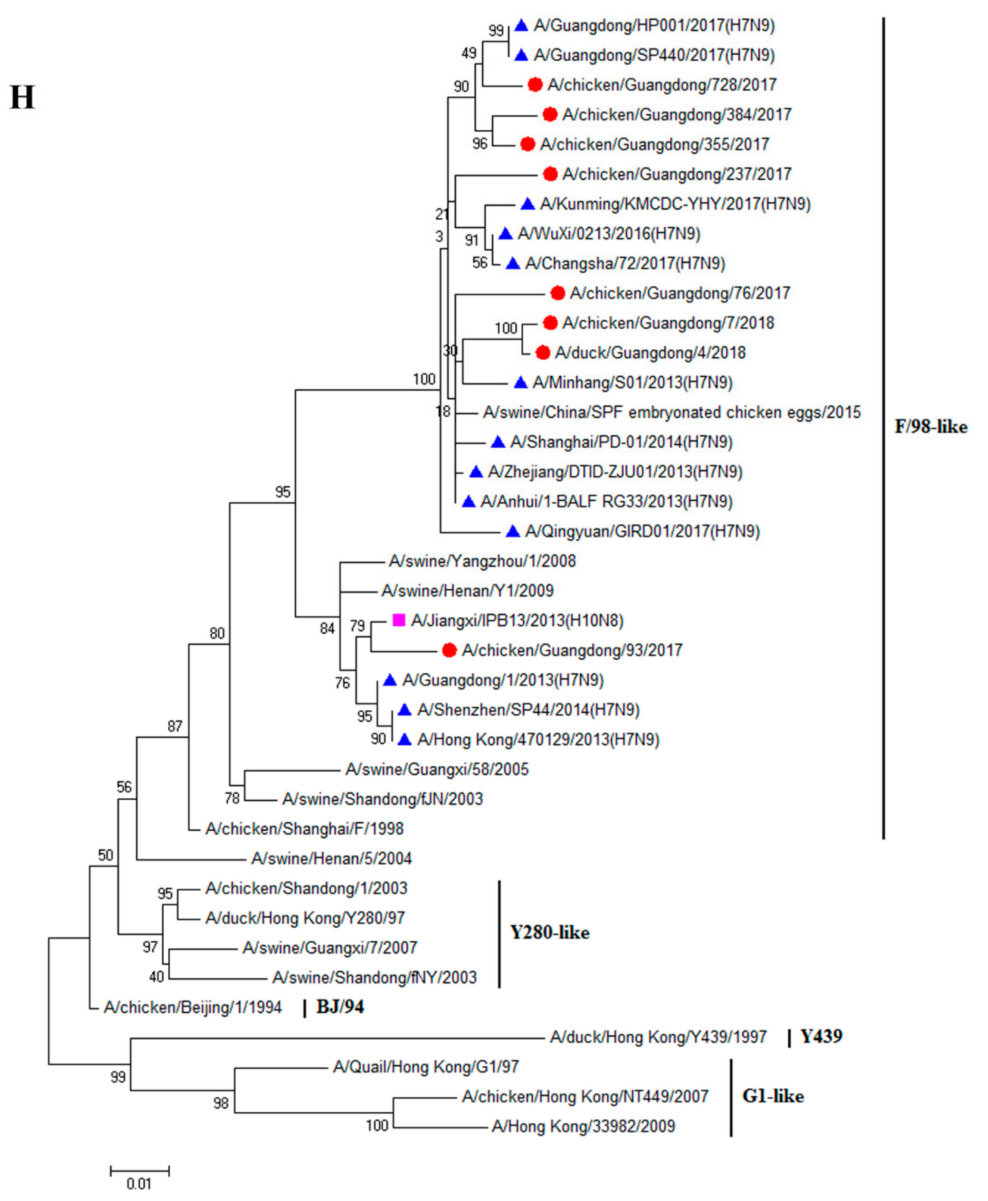
Phylogenetic analysis of eight H9N2 influenza viruses. (**A**) Phylogenetic trees of HA genes based on nucleotides (nt) 34 to 1716. (**B**) Phylogenetic trees of NA genes based on nt 20 to 1420. (**C**) Phylogenetic trees of PB2 genes based on nt 28 to 2307. (**D**) Phylogenetic trees of PB1 genes based on nt 25 to 2298. (**E**) Phylogenetic trees of PA genes based on nt 25 to 2175. (**F**) Phylogenetic trees of NP genes based on nt 46 to 1542. (**G**) Phylogenetic trees of M genes based on nt 26 to 1007. (**H**) Phylogenetic trees of NS genes based on nt 27 to 864. Solid red circles indicate H9N2 avian influenza viruses isolated in this study, solid blue triangles indicate H7N9 viruses, and solid pink squares indicate H10N8 viruses. The sequences were aligned by the Clustal W and Maximum Likelihood method (ML), trees were generated with 1000 bootstrap replicates using MEGA 7.

Finally, eight H9N2 viruses were successfully isolated from swabs collected during 2017–2018, CK7/18, DK4/18, CK76/17, CK93/17, CK237/17, CK355/17, CK384/17, and CK728/17. The nucleotide sequences were deposited in the GenBank (the accession numbers are MN384765–MN384772, MN384773–MN384780, MN384723–MN384730, MN385406–MN385413, MN385368–MN385375, MN385376–MN385383, MN385385–MN385392, MN385393–MN385400, respectively). To reveal the genetic relationship and ecology of the H9N2 viruses in South China between 2017 and 2018, the full genomes of eight strains were sequenced. The homological and phylogenetic analysis showed that the HA genes of DK4/18 and the other 7 viruses shared 93.6–99.9% similarity at the nucleotide level and 93.9–100% similarity at the amino acid level, and belonged to the h9.4.2.5 clade and Y280-like lineage (Figure 1A). These eight HA genes obtained a conserved amino acid sequence of PSRSSR↓GL at the cleavage site. In this study, the amino acids in the HA protein that prompted virus adaption to mammals were 101Y, 139T, 153W, 183N, 194L, 195Y, 196T, 225G, 226L, 227M, 228G, and 321L. Except for CK76/17 with 155I and 212V, the remaining viruses were T at 155 and I at 212. Except for CK355/17 with 190 A, the other seven viruses were T at 190. Except for CK7/18, DK4/18 and CK76/17 (CK7/18 and DK4/18 with 149 K and CK76/17 with 149S), the other five viruses were R at 149 (Table 1).

The neuraminidase (NA) genes of DK4/18 and the other 7 viruses shared 93.2–99.4% similarity at the nucleotide level and 93.8–99.6% similarity at the amino acid level, and belonged to the Y280-like lineage (Figure 1B). Three amino acids (63–65) were deleted at the NA stalk region of these viruses; the polymerase basic protein 2 (PB2) genes were 92.2–99.1% similar at the nucleotide level, belonged to the Y439-like lineage (Figure 1C), and were close to the PB2 of the recently circulating H7N9 viruses. The mammalian-adjusted amino acids in the PB2 protein were 253D, 292V, 591Q, 627E, 685G, and 701D. The amino acids at 588 of CK7/18, CK93/17, CK355/17, and CK384/17 were V, and those of other viruses were I, A, or T (Table 1). The polymerase basic protein 1 (PB1) genes shared 94.1–100% similarity at the nucleotide level, belonged to the F/98-like lineage (Figure 1D) and were similar to the PB2 of the recently circulating H7N9 viruses. The amino acid at 368 in the PB1 protein of these eight viruses was V (Table 1). The polymerase acidic protein (PA) genes shared 95.0–99.9% similarity at the nucleotide level and belonged to the F/98-like lineage (Figure 1E). Except for the PA of CK76/17, which was similar to that of the H7N9 that circulated in 2013, the PA of the remaining viruses was close to those of the currently circulating H7N9. The amino acid at 356 in the PA protein of these eight viruses was R (Table 1). The nucleoprotein (NP) genes shared 93.5–99.4% similarity at the nucleotide level and belonged to the F/98-like lineage (Figure 1F). Except for the NP of CK76/17, which was similar to that of the H7N9 that circulated in 2013–2014, the NPs of the remaining viruses were close to those of the recently circulating H7N9 virus. The M genes shared 96.1–99.7% similarity at the nucleotide level, belonged to the G1-like lineage (Figure 1G) and were similar to H10N8. The nonstructural protein (NS) genes shared 94.0–99.5% similarity at the nucleotide level and belonged to the F/98-like lineage (Figure 1H). The NS of CK93/17 was close to that of H7N9 and H10N8, while the NS of the others were similar to the recently circulating H7N9. These viruses belong to a new genotype, designated as G57.

### 3.2. H9N2 Viruses Effectively Replicate In Vitro

**Figure 2:**
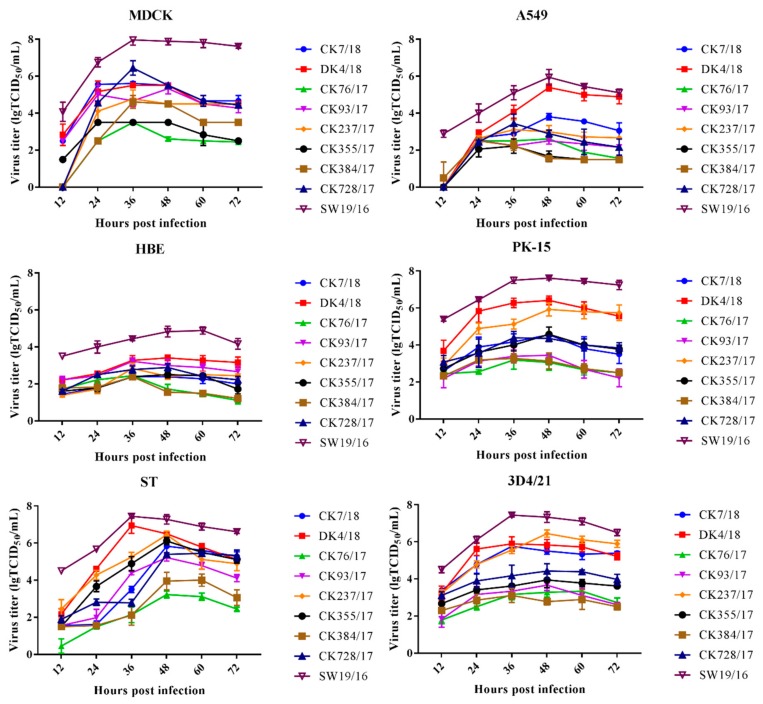
Replication kinetics of the eight H9N2 influenza viruses in different cells culture. Replication kinetics of viruses in Madin-Darby canine kidney (MDCK) cells inoculated with virus at a multiplicity of infection (MOI) of 0.001, in A549, HBE, PK-15, ST, and 3D4/21 cells inoculated with virus at an MOI of 0.01. A strain of H1N2 swine influenza virus, SW19/16, was used as control. The data of each time point indicates the mean ± standard deviation of three independent experiments.

To characterize the replication capacity of the H9 AIVs in vitro, the growth kinetics of the H9N2 viruses were determined by infecting MDCK, A549, HBE, PK-15, ST, and 3D4/21 cells. The SW19/16 virus was used as a control. In MDCK cells, the peak titer of CK728/17 was higher than that of CK7/18, DK4/18, CK76/17, CK93/17, CK237/17, CK355/17, and CK384/17 (*P* < 0.05). The peak titer of CK7/18 was higher than that of CK76/17, CK237/17, CK355/17, and CK384/17 (*P* < 0.05). The peak titer of DK4/18 was higher than that of CK76/17, CK355/17, and CK384/17 (*P* < 0.05). The peak titer of CK93/17 was higher than that of CK76/17 and CK355/17 (*P* < 0.001). The peak titer of CK237/17 was higher than that of CK76/17 and CK355/17 (*P* < 0.001). The peak titer of CK384/17 was higher than that of CK76/17 and CK355/17 (*P* < 0.01) (Figure 2).

In A549 cells, the peak titer of DK4/18 was higher than that of CK7/18, CK76/17, CK93/17, CK237/17, CK355/17, CK384/17, and CK728/17 (*P* < 0.001). The peak titer of CK7/18 was higher than that of CK76/17, CK93/17, CK355/17, and CK384/17 (*P* < 0.01). The peak titer of CK728/17 was higher than that of CK355/17 and CK384/17 (*P* < 0.05) (Figure 2). 

In HBE cells, the peak titer of DK4/18 was higher than that of CK7/18, CK76/17, CK237/17, CK355/17, CK384/17, and CK728/17 (*P* < 0.05). The peak titer of CK93/17 was higher than that of CK7/18, CK76/17, CK355/17, and CK384/17 (*P* < 0.05). The peak titer of CK728/17 was higher than that of CK7/18, CK76/17, and CK384/17 (*P* < 0.05).

In PK-15 cells, the peak titer of DK4/18 was higher than that of CK7/18, CK76/17, CK93/17, CK355/17, CK384/17, and CK728/17 (*P* < 0.001). The peak titer of CK237/17 was higher than that of CK7/18, CK76/17, CK93/17, CK355/17, CK384/17, and CK728/17 (*P* < 0.001). The peak titer of CK355/17 was higher than that of CK76/17, CK93/17, and CK384/17 (*P* < 0.01). The peak titer of CK7/18 was higher than that of CK76/17, CK93/17, and CK384/17 (*P* < 0.01). The peak titer of CK728/17 was higher than that of CK76/17, CK93/17, and CK384/17 (*P* < 0.05). (Figure 2).

In ST cells, the peak titer of DK4/18 was higher than that of CK7/18, CK76/17, CK93/17, CK384/17 and CK728/17 (*P* < 0.01). The peak titer of CK237/17 was higher than that of CK76/17, CK93/17, CK384/17 and CK728/17 (*P* < 0.05). The peak titer of CK355/17 was higher than that of CK76/17, CK93/17 and CK384/17 (*P* < 0.05). The peak titer of CK7/18 was higher than that of CK76/17 and CK384/17 (*P* < 0.001). The peak titer of CK728/17 was higher than that of CK76/17 and CK384/17 (*P* < 0.001). The peak titer of CK93/17 was higher than that of CK76/17 and CK384/17 (*P* < 0.01) (Figure 2). 

In 3D4/21 cells, the peak titer of CK237/17 was higher than that of CK76/17, CK93/17, CK355/17, CK384/17, and CK728/17 (*P* < 0.001). The peak titer of DK4/18 was higher than that of CK76/17, CK93/17, CK355/17, CK384/17, and CK728/17 (*P* < 0.001). The peak titer of CK7/18 was higher than that of CK76/17, CK93/17, CK355/17, CK384/17 and CK728/17 (*P* < 0.01). The peak titer of CK728/17 was higher than that of CK76/17 and CK384/17 (*P* < 0.05). (Figure 2).

In MDCK, HBE and PK-15 cells, the peak titer of SW19/16 was higher than that of CK7/18, DK4/17, CK76/17, CK93/17, CK237/17, CK355/17, CK384/17, and CK728/17 (*P* < 0.05). In A549 and ST cells, the peak titer of SW19/16 was higher than that of CK7/18, CK76/17, CK93/17, CK237/17, CK355/17, CK384/17, and CK728/17 (*P* < 0.05). In 3D4/21 cells, the peak titer of SW19/16 was higher than that of CK7/18, DK4/17, CK76/17, CK93/17, CK355/17, CK384/17, and CK728/17 (*P* < 0.05) (Figure 2).

### 3.3. H9N2 Viruses Preferentially Bind to the α-2,6-linked SA Receptor

**Figure 3:**
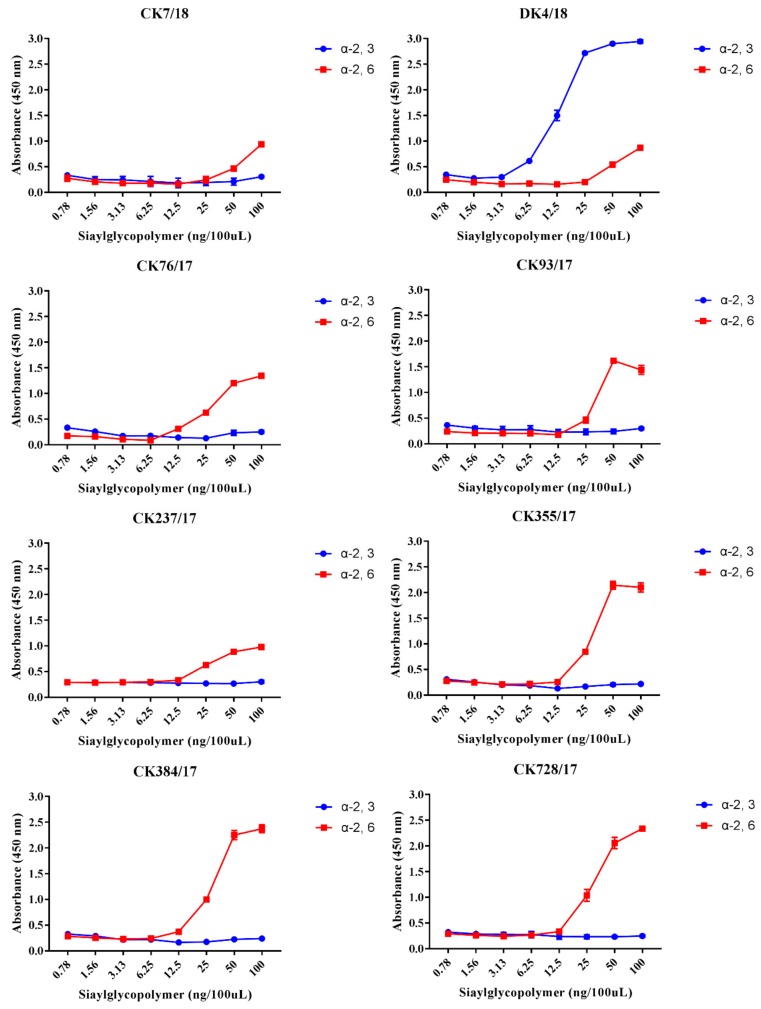
Receptor binding properties of the 8 H9N2 influenza viruses. Solid-phase binding assays for CK7/18, DK4/18, CK76/17, CK93/17, CK237/17, CK355/17, CK384/17, and CK728/17 to two different sialylglycopolymers (3’- SLN colored in blue and 6’- SLN colored in red).

To characterize the receptor-binding affinity of these eight H9N2 AIVs, a solid-phase binding assay was performed. The results indicate that viruses CK7/18, CK76/17, CK93/17, CK237/17, CK355/17, CK384/17, and CK728/17 strongly and exclusively bind to α-2,6-siaylglycopolymer (human receptor), while not binding to the 3-siaylglycopolymer (avian receptor). The virus DK4/18 showed stronger binding affinity to α-2,6-siaylglycopolymer and α-2,3-siaylglycopolymer (Figure 3).

### 3.4. Virus Replication in the Lung of Mice

**Figure 4:**
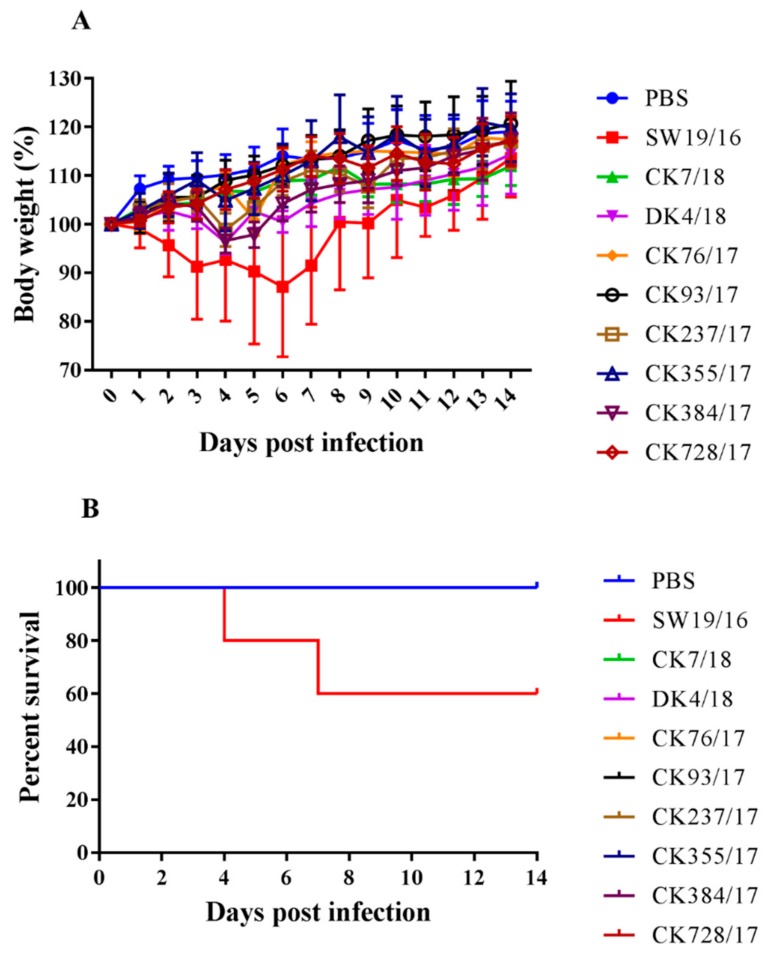
Variation of body weight (**A**) and survival (**B**) of mice inoculated with viruses. Eighty 6-week-old female BALB/c mice were randomly divided into ten groups. Each group contains eight mice. One group of mice were anesthetized with isoflurane and intranasally inoculated with 100 μL PBS as a negative control. One group of mice were anesthetized and intranasally inoculated with SW19/16, at a dose of 10^5^ EID_50_, in 100 µL. The other eight groups of mice were anesthetized and intranasally inoculated with corresponding H9N2 viruses, respectively, at a dose of 10^6^ EID_50_, in 100 µL. In each group, three mice were euthanized and brains, spleens, kidneys, and lungs were collected at 3 dpi. The remaining mice were continually weighed until 14 dpi.

To detect the replication, pathogenicity of these eight H9N2 viruses in mice, 6-week-old female SPF mice were intranasally infected with corresponding viruses at a dose of 10^6^ EID_50_, respectively. The SW19/16 virus was used as swine influenza virus control at a dose of 10^5^ EID_50_. DK4/18, CK237/17, and CK384/17 caused up to 6.88% body weight loss. SW19/16 caused up to 29.58% body weight loss and caused 40% mice death (Figure 4A,B). No virus was detected in brains, spleens, or kidneys in all groups. SW19/16, DK4/18, CK237/17, and CK728/17 could replicate in the lung with a titer of 5.83 ± 0.42 lgEID_50_/g/mL, 5.50 ± 0.20 lgEID_50_/g/mL, 1.08 ± 1.53 lgEID_50_/g/mL, and 0.58 ± 0.82 lgEID_50_/g/mL, respectively (Table 2). All mice infected with viruses seroconverted with titers of 1:20–1:640 (Table 3).

### 3.5. Virus Replication in the Turbinate of Pigs and Virus Shedding Last for Four Days

**Figure 5:**
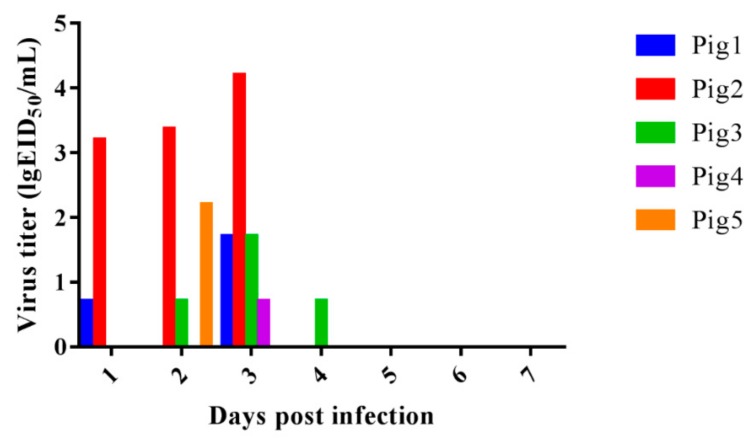
Virus shedding of H9N2 influenza virus in inoculated pigs. Thirteen healthy 3-week-old pigs were randomly divided into three groups. Of these, five pigs were intranasally inoculated with CK93/17 at a dose of 10^7^ EID_50_ in 1 mL as treatment group. Three pigs were intranasally inoculated with 1 mL PBS as control group. One-day post-inoculation (1 dpi), five pigs were housed with treatment pigs as a physical contact group. Nasal wash was collected from all pigs from 1 to 10 dpi and titrated in 9 to 11-day-old embryonated eggs. Two treatment pigs, one contact pig, and one control pig were euthanized at 3 dpi. One treatment pig, two contact pigs, and one control pig were euthanized at 5 dpi. The viral titers in tissues were titrated in 9 to 11-day-old embryonated eggs.

To detect the replication, pathogenicity, and transmission of these eight H9N2 viruses, 3-week-old pigs that confirmed negative for influenza, brucellosis, and pseudorabies were intranasally inoculated with CK93/17 at a dose of 10^7^ EID_50_ in 1 mL. We observed no obvious clinical symptoms during the experiment. Virus shedding was detected from all treatment pigs with a titer of 0–4.2 lgEID_50_/mL. The virus shedding titer reached a peak at 3 dpi (4/5), with a value of 4.2 lgEID_50_/mL. Virus shedding could be detected in neither the physical contact pigs nor the control pigs. Virus replication was only detected in turbinate (2/2) at 3 dpi with a titer of 1.37 lgEID_50_/g/mL (Figure 5). The HI results indicate that one of two pigs seroconverted into positive, with titers of 1:40 and 1:160 at 7 and 14 dpi, respectively (Table 4).

## 4. Discussion

In this study, the eight viruses isolated during 2017–2018 in South China belonged to the recently confirmed Genotype G57 [11,12]. These viruses belonged to the Y280-like lineage, with similar internal genes to recently circulating H7N9 viruses, which have been predominant in China [7,12]. All results revealed that H9N2 viruses had been dramatically evolving and underwent complicated reassortment with H7N9 viruses and that these viruses preferentially bind to the human-like receptor and can effectively replicate in mammalian cells and pigs. 

The H9N2 viruses used in the present study efficiently replicated in MDCK cells, which is consistent with previous studies [7,8]. With an inoculation dose of 0.2 MOI, the viral titers of some G1-like and Eurasian wild-type reassortant H9N2 viruses were 2.3–4.9 lgTCID_50_/mL and the detectable and peak titers appeared at 16 hpi and 32 hpi, respectively [42]. When the inoculation dose was 0.001 MOI, the peak viral titer of the G1-like virus appeared at 36 hpi [43]. In this study, the detectable viral titers of CK7/18, DK4/18, CK93/17, and CK355/17 appeared at 12 hpi when MDCK cells were inoculated with viruses at a dose of 0.001 MOI. The viral titers of CK355/17 and CK93/17 peaked at 24 and 48 hpi, respectively. The peak viral titers of the remaining viruses appeared at 36 hpi, and the maximum viral titers were 3.50–5.61 lgTCID_50_/mL, which were higher than those of G1-like viruses [42]. Besides the condition of cell culture affecting infectivity and growth curve of viruses, the discrepancies in the replication efficiency of those viruses in MDCK cells might be due to the inoculation dose and the special characteristics of the viruses themselves. The choice of MOI could affect infection and spread of the viruses in these cells [44]. 

H9N2/Y280, H9N2/G9, and G57 genotype viruses exhibited poor replication in A549 cells when the inoculation dose was 0.01 MOI [7,8,45]. In contrast, the H9N2/G1 virus can efficiently replicate in A549 cells with titers of 4.6 and 5.5 lgTCID_50_/mL at 24 and 48 hpi, respectively [7]. The eight viruses isolated in the present study also displayed effective replication in A549 cells, even though they did not belong to G1-like lineage viruses. Interestingly, the viral titers of DK4/18 showed similarity to those of H9N2/G1 [7]. That lysine mutated into arginine at the 356 amino acid position (K356R) in the PA protein of the H9N2 AIV could increase the activity of polymerases in A549 cells [46]. All eight H9N2 AIVs in the present study already acquired such a mutation at the 356 positions of PA, which would enable these to replicate in A549 cells.

At an MOI of 1, the peak viral titer of H1N1pdm can reach 4 lgTCID_50_/mL in HBE cells [47]. At an MOI of 0.01, the peak viral titers of H9N2 viruses were 2.39–3.41 lgTCID_50_/mL. It indicated that H9N2 influenza viruses recently circulating in South China effectively replicate in HBE cells and these viruses pose a potential risk to infect humans.

The growing peak titer of H6 subtype influenza viruses isolated from the environment in A549 and MDCK were higher than that in PK-15 cells [48]. Unlike H6 viruses, these H9N2 AIVs grew better in PK-15 cells than in A549. The viral titers of CK7/18, CK76/17, CK93/17, CK384/17, and CK728/17 in MDCK were 2.0–114.8-fold higher than those in PK-15 cells, while the titers of DK4/18, CK237/17, and CK355/17 in PK-15 were 8.1–13.9-fold higher than those in MDCK. The viruses exhibited different growth features, and this kind of phenomenon has also been found in ST and 3D4/21 cells; which might account for the discrepancy of receptor distribution and secretion of the enzyme in the different cells. Mutations in the PB2 gene play an important role in favoring AIVs to adapt to mammalian hosts. H9N2 viruses harboring PB2-588 V exhibited higher polymerase activity in 293T and MDCK cells [49], and 292V could also increase the replication of AIVs in human cells [50]. All eight H9N2 viruses carried PB2 with 292V. Additionally, CK7/18, CK355/17, and CK384/17 carried 588 V. These mutations might be involved in favoring viruses to effectively replicate in MDCK, A549, and pig cells. Further genetic experiments are needed to clarify the function of the 292V with 588 V combination and how this affects replication of avian viruses in mammalian cells.

Receptor-binding preference was important for the influenza virus to replicate and transmit [51]. 226L is a critical motif for viral binding affinity for α-2,6-linked sialic acid receptors and efficient replication in mammalian hosts [52,53]. The amino acids at positions 226L of all eight viruses were conserved, and all but the DK4/18 virus preferentially bound to the human receptor, which is consistent with previous studies [54]. The DK4/18 virus had a greater binding affinity for the α-2,3-linked sialic acid receptor than to the a2,6-linked sialic acid receptor, even though it carried 226L. A possible reason for this is that the preferential receptor binding feature is decided by multiple amino acids and that 226L plays an important but not decisive role. The mutations I155T, H183N, and A190T favor H9N2 virus binding to the human-type receptor [53,55,56]. Except for CK76/17 with I at 155 and CK237/17 with A at 190, the amino acids of the remaining viruses were 155T, 183N, and 190T. These data identify eight viruses with great affinity to bind to the human receptor, which is also supported by the solid-phase ELISA results. 

Consistent with previous studies [57,58], H9N2 viruses caught minor bodyweight decrease and did not cause death in the mice model. The viral titers can reach 4–6 lgEID_50_/mL in the lung of mice infected with duck-origin H9N2 viruses [57,58]. In our study, the viral peak titer of DK4/18 in the lung of mice reached 5.5 lgEID_50_/g/mL and it is higher than that of chicken-origin viruses. Whether the virulence of duck-origin H9N2 viruses to mammals is stronger than that of chicken-origin ones needs a lot more studies to address in the future.

Pigs have been considered as intermediate hosts and “mixing vessels” that favor influenza viruses to acquire adaptation to humans, thereby increasing the risk of a pandemic [59,60,61]. In an inoculation dose of 10^7^ PFU, detectable virus shedding started as early as 1 to 2 dpi and lasted for 5–6 days from pigs inoculated with G1-like and Y280-like viruses [62]. In the present study, virus shedding of pigs inoculated with CK93/17 in a dose of 10^7^ lgEID_50_ could be detected from 1 to 4 dpi. The time of virus shedding was shorter than that of those G1-like and Y280-like viruses. Besides viruses belonging to different genotypes, the difference in inoculation dose might account for this discrepancy. Similar to pigs inoculated with A/quail/Hong Kong/G1/1997, the virus shedding could be detected from contact pigs (1/4) at 5 dpi [63]. However, in our study, we failed to detect virus shedding by contact pigs. The infection manner in this study was intranasal inoculation, but in the previous study, the infection method was intratracheal inoculation, which might account for the differences in findings. Consistent with previous research [62,64], clinical signs were observed neither in treatment pigs nor contact pigs during the experiment. The antibody titer from pigs inoculated with G1-like and Y439-like duck viruses (isolated in 2009) were 1:320–1:640 [64], which is higher than that of pigs inoculated with CK93/17. This discrepancy might be due to either the use of different strains or differences in the susceptibilities of the HI and ELISA assays.

Taken together, a novel H9N2 genotype recently circulating in poultry preferentially bound to the human receptor and acquired multiple mutations adaptive to mammals. Efforts to strengthen the surveillance of H9N2 AIVs and illuminate their pathogenicity are urgently needed to assess the potential risk of H9N2 AIVs to public health. 

## Figures and Tables

**Table 1:** The molecular characteristics of the H9N2 influenza viruses used in this study.

Viruses	Cleavage Site in HA	Residues in HA (H3 Numbering)					Residues in PB2							Residues in PB1	Residues in PA
		I155T	H183N	A190T/V	T212I	Q226L	D253N	I292V	588V	591K	E627K	G685R	D701N	I368V	K356R
A/chicken/Guangdong/7/2018	PSRSSR	T	N	T	I	L	D	V	V	Q	E	G	D	V	R
A/duck/Guangdong/4/2018	PSRSSR	T	N	T	I	L	D	V	I	Q	E	G	D	V	R
A/chicken/Guangdong/76/2017	PSRSSR	I	N	T	V	L	D	V	A	Q	E	G	D	V	R
A/chicken/Guangdong/93/2017	PSRSSR	T	N	T	I	L	D	V	V	Q	E	G	D	V	R
A/chicken/Guangdong/237/2017	PSRSSR	T	N	A	I	L	D	V	T	Q	E	G	D	V	R
A/chicken/Guangdong/355/2017	PSRSSR	T	N	T	I	L	D	V	V	Q	E	G	D	V	R
A/chicken/Guangdong/384/2017	PSRSSR	T	N	T	I	L	D	V	V	Q	E	G	D	V	R
A/chicken/Guangdong/728/2017	PSRSSR	T	N	T	I	L	D	V	I	Q	E	G	D	V	R

**Table 2:** Virus titer in different organs of mice.

Virus	Lung	Spleen	Kidney	Brain
SW19/16	5.83 ± 0.42	ND	ND	ND
CK7/18	ND	ND	ND	ND
DK4/18	5.50 ± 0.20	ND	ND	ND
CK76/17	ND	ND	ND	ND
CK93/17	ND	ND	ND	ND
CK237/17	1.08 ± 1.53	ND	ND	ND
CK355/17	ND	ND	ND	ND
CK384/17	ND	ND	ND	ND
CK728/17	0.58 ± 0.82	ND	ND	ND
PBS	ND	ND	ND	ND

Note: The virus titers (lgEID_50_/g/mL) were expressed as mean ± standard deviation. ND: indicate that the titers were under detectable.

**Table 3:** HI titers of sera collected from mice inoculated with influenza viruses.

Virus	Mouse 1	Mouse 2	Mouse 3	Mouse 4	Mouse 5
SW19/16	80	160	80	80	80
CK7/18	160	160	160	80	80
DK4/18	320	320	320	640	320
CK76/17	80	80	80	80	80
CK93/17	80	40	40	40	20
CK237/17	80	160	160	320	160
CK355/17	80	80	80	80	40
CK384/17	160	20	40	160	160
CK728/17	160	320	160	160	160
PBS	<10	<10	<10	<10	<10

**Table 4:** Seroconversion of pigs inoculated with or exposed to H9N2 influenza viruses.

dpi.	Inoculated Pigs					Physical Contact Pigs					Control Pigs		
	Pig 1	Pig 2	Pig 3	Pig 4	Pig 5	Pig 6	Pig 7	Pig 8	Pig 9	Pig 10	Pig 11	Pig 12	Pig 13
7	-	-	40	-	<10	<10	<10	-	-	-	-	<10	-
14	-	-	160	-	<10	<10	<10	-	-	-	-	<10	-

Note: “-” indicate that the pigs were sacrificed at 3 dpi. (dpc.) or 5 dpi. (dpc.).
